# Predictors of Cancer Detection Behaviors Among Older Nigerian Men

**DOI:** 10.1093/geroni/igaa057.1060

**Published:** 2020-12-16

**Authors:** Kelsey Brodie, Darlingtina Esiaka, Candidus Nwakasi

**Affiliations:** 1 Union College, Schenectady, New York, United States; 2 Union College, Union College, New York, United States; 3 University of Southern Indiana, Evansville, Indiana, United States

## Abstract

Recent research emerging from Nigeria suggest an increasing mortality due to cancer, especially among older Nigerian men who are at a higher risk of being diagnosed at fatal or advanced stage of cancer. With older age as a significant risk factor for cancer development such as prostate cancer in men, this study explored factors that influence cancer detection behavior among aging Nigerian men. Specifically, we examined possible predictors of current and future intentions to engage in early cancer detection behaviors among Nigerian men. Participants (N=143), with a mean age of 44.73 (SD = 6.15), responded to measures assessing health (cancer detection behaviors), social (masculinity, self-esteem, attachment), and psychological (active coping) factors. Demographic and ecological questions were also included in the survey. Results revealed that education, masculinity, and anxious attachment were significant predictors of current cancer detection behavior. Education, masculinity, and anxious attachment also predicted future cancer screening intentions. We discuss the implication of result for health policy, health education and cancer prevention interventions for Nigerian men and for the global campaign for early cancer detection.

